# Impact of a nutrition consultation on the rate of high output stoma-related readmission: an ambispective cohort study

**DOI:** 10.1038/s41598-021-96136-7

**Published:** 2021-08-17

**Authors:** Manuela Moreno Santamaría, José Javier Arenas Villafranca, Jimena Abilés, Francisco Rivas Ruiz, Pilar Utrilla Navarro, Begoña Tortajada Goitia

**Affiliations:** 1 Pharmacy and Nutrition Department, Costa del Sol Hospital, A-7, Km 187, 29603 Marbella, Málaga Spain; 2 Research Department, Costa del Sol Hospital, A-7, Km 187, 29603 Marbella, Málaga Spain; 3 Pharmacology Department, University of Granada, Granada, Spain

**Keywords:** Health care, Gastrointestinal diseases

## Abstract

The aims of this study were to assess the impact of a follow-up nutrition consultation for ostomy patients on the rate of high output stoma (HOS)-related readmissions, as well as on the detection of poor nutritional status and their management, and to determine the associated economic impact. A single-centre ambispective cohort study was conducted in which all adult patients undergoing intestinal resection and stoma creation were recruited. Two nutrition consultations were established for early follow-up after hospital discharge and patients were prospectively included. Additionally, a retrospective search was carried out to include a control group. In both groups, a 12-month follow-up was conducted to record readmissions associated with high output stoma. A multivariate logistic regression was performed. Statistical significance level was established at *p* < 0.05. 170 patients were recruited, 85 patients in each cohort. Demographic data and clinical characteristics were recorded. A significant difference was observed in HOS-related readmissions, with readmission rates of 28.6% vs 10.3% in the retrospective and prospective cohort, respectively. At the first follow-up consultation, 50.5% of patients presented some degree of protein-calorie malnutrition. A statistically significant improvement in nutritional status was observed in the second evaluation. The intervention carried out resulted in a total saving of €24,175. Early follow-up of patients after discharge resulted in a significant reduction in the rate of HOS-related readmissions and allowed to identify a high percentage of patients with malnutrition. The cost analysis showed the process to be a cost-effective improvement.

## Introduction

Defunctioning stoma formation following bowel resection is a procedure commonly associated with complications and increased morbidity in patients undergoing colorectal surgery^1,2^. These complications occur in 70–80% of patients^1,3^ and include skin problems, necrosis, obstruction, stoma retraction, stoma prolapse, and parastomal herniation, but there are other complications that have been less studied, such as electrolyte disturbances, acute renal failure, and malnutrition associated with the development of high output stoma (HOS)^4,5^.

Dehydration and malnutrition secondary to HOS in ileostomy patients are associated with increased hospital readmission rates and significantly longer hospital stays, and account for the highest percentage of readmissions after colorectal surgery^4,6,7^. Although HOS has been described as a less common complication in colostomy patients given that a large portion of the small bowel and the colon are retained, thus helping water resorption, its occurrence cannot be ruled out^5^.

HOS patient readmission rates, which represent 20–30% of the total rate of readmissions due to ostomy complications^8^, their increased morbidity^7^, and the resulting health resource utilization have prompted a number of authors to developed tools to identify risk factors and elaborate interventions to avoid such complications and make their management easier^9,10^. Perioperative follow-up studies of ostomy patients conducted by multidisciplinary teams^5,11,12^ have described health education strategies such as ostomy education, stoma output monitoring, and clinical and pharmacological management of HOS as primary interventions^10,13^. However, despite advances in the identification of the population at risk and the determination of appropriate measures to reduce readmission rates due to dehydration, there is limited evidence for their effectiveness. Studies assessing the usefulness of their implementation are needed^10^.

In addition to electrolyte disturbances, nutritional compromise secondary to chronic HOS has an impact on these patients’ health^5^. The consequences of a poor nutritional status on health resource utilization and mortality rate highlight the importance of implementing procedures for its accurate diagnosis and the provision of appropriate nutritional support in the standard clinical practice^14,15^.

The primary objective of our study was to assess the impact of a nutrition and stoma output monitoring consultation for patients who had recently undergone ostomy surgery on the rate of readmissions for HOS and the detection of poor nutritional status and their management. The secondary objective was to determine the financial impact of this intervention.

## Methods

A single-centre ambispective cohort study was conducted in a 350-bed hospital to evaluate the impact of a follow-up nutrition consultation for ostomy patients following hospital discharge on the rate of readmissions associated with HOS. A protocol was established for patient follow-up in the nutrition clinic, enrolling patients prospectively and identifying patients retrospectively for use as a control group.

### Patient population

All patients were screened consecutively for both the retrospective and the prospective cohorts based on predefined inclusion and exclusion criteria until completing the sample. The inclusion criteria were: patients aged ≥ 18 years having undergone a bowel resection with defunctioning stoma formation. The exclusion criteria were: patients who already had a stoma or who had undergone a bowel resection prior to the date of inclusion, and patients having been hospitalized in the intensive care unit for more than seven days following surgery.

Sample size was calculated for a power of 80%, a 95% confidence level, and a 1:1 new consultation exposure ratio (with respect to the retrospective cohort). A sample size of 61 patients in each cohort was estimated and increased to 85 per cohort assuming a 25–35% attrition rate.

### Retrospective study

From March 2012 to July 2014, a retrospective review of the data from the site’s electronic medical records was performed.

### Prospective study

From June 2016 to March 2018, all patients were included in the follow-up process involving an assessment visit soon after hospital discharge and a second visit one month later, as follows:

- The first follow-up visit took place 7–10 days after hospital discharge. During this consultation, the patient’s nutritional history was obtained, the nutritional status assessed, and the dietary progression evaluated. Based on our hospital’s standard protocol^16^, patients were instructed on how to identify symptoms associated with dehydration, monitor their stoma output, and manage any related problem that might occur in this respect (Appendix 1).
- The second follow-up visit took place one month later, with a further assessment of the nutritional status and dietary progression, and reporting any occurrences of high output stoma associated with the ostomy.

In both visits, nutritional diagnosis was established based on data collected from the medical records and the patient interview, changes in weight, dietary progression, and laboratory parameters. Weight variation was calculated based on the difference between current weight and weight recorded three months prior to the surgical intervention (usual weight). The following nutritional diagnoses were determined using the Subjective Global Assessment tool: good nutritional status, nutritional risk, and malnutrition. They were further described as mild, moderate or severe according to severity^17^. Patients at nutritional risk or with some degree of malnutrition were advised on how to optimize their diet and reach appropriate nutritional requirements to recover a good nutritional status.

### Follow-up and endpoint assessment in the retrospective and prospective studies

For both studies, demographic characteristics (age, sex), comorbidities (hypertension, renal disease, diabetes, dyslipidaemia), underlying disease leading to the surgical intervention, type of surgery (urgent, elective), length of resection and type of stoma, presence of high output stoma after surgery, and length of hospital stay were collected from electronic medical records. Mortality was recorded from the Spanish National Death Index^18^.

High output stoma-related readmissions in the 12 months following post-surgery discharge and subsequent length of hospital stay were recorded.

### Economic impact assessment

The economic impact assessment was carried out with data collected from the Andalusian Public Health System (*Sistema Sanitario Público Andaluz*, Spain) based on the consumer price index (CPI) for 2019. The cost of the new consultation and an estimate of the cost of the hospital stay for HOS-related readmission were included.

The cost of the hospital stay following readmission for both cohorts was calculated using the total number of days of hospital stay in each medical specialty and their unit cost per day of hospitalization (direct and indirect costs): Internal Medicine Unit (€340), Surgery Unit (€406), Emergency Unit (€155).

The estimation of the cost of the nutrition consultation was based on the cost of the nutritional assessment calculated using the cost of one minute of work of a nutritionist, based on their gross salary (€28,994) and assuming a working day of 7.5 h/day and 220 working days/year. The unit cost of a nutritionist was therefore estimated at €0.29/minute of work. The estimated time required to perform a comprehensive nutritional assessment during the first visit was 40 min/patient (€11.6). For the second visit, the time required was estimated at 20 min/patient (€5.8).

### Statistics

This was an ambispective study: a descriptive analysis was performed using measures of central tendency, dispersion and position for quantitative variables, and frequency distribution for qualitative variables. The cumulative incidence of high output was calculated at 6 and 12 months according to the cohort, estimating the odds ratio (OR) for the retrospective cohort and the corresponding 95% confidence interval (95% CI). To assess differences between cohorts, a chi-square test was used (or Fisher’s exact test for expected frequencies under 5) for qualitative variables, and Student t test (or Mann–Whitney U test for non-normal distributions) for quantitative variables. Finally, a multivariate logistic regression was performed taking HOS-related readmissions in 12 months as the outcome variable and incorporating, together with the cohort variable, the unbalanced variables found in a prior bivariate analysis, obtaining ORs with their appropriate 95% CIs. The statistical significance level was set at *p* < 0.05.

The descriptive analysis using measures of central tendency, dispersion, and position for quantitative variables, and frequency distribution for qualitative variables was performed only for the prospective study. Paired differences were evaluated at two time points using Student t test for paired quantitative variables or McNemar test for paired qualitative variables. The statistical significance level was set at *p* < 0.05. The statistical software used was *SPSS Statistics 26.0*.

### Ethics

The study protocol was carried out according to the guidelines established by the Declaration of Helsinki and was approved by the Hospital Costa del Sol Ethics Committee for Clinical Research: 31/03/2016/007_Mar16_RelOAD. All study patients granted their informed consent in writing to participate in the study.

## Results

The study population was composed of 170 consecutively enrolled patients, 85 in each cohort. The corresponding demographic data are shown in Table 1.

**Table 1:** General characteristics of the study population (N = 170).

	Retrospective cohort (N = 85)		Prospective cohort (N = 85)		*p*
	n	%	n	%	
**Age** (years) (mean-SD)	59.4	16.2	64.3	13.1	0.032
**Sex**					
Male	39	45.9	62	72.9	0.001
Female	46	54.1	23	27.1	
**Comorbidities**					
Hypertension	27	31.8	43	50.6	0.019
Renal disease	2	2.4	3	3.5	1
Diabetes	11	12.9	19	22.4	0.159
Dyslipidaemia	15	17.6	33	38.8	0.004
**Underlying disease**					
Malignant	61	71.8	70	82.4	0.144
Benign	24	28.2	15	17.6	
**Cancer therapy**^a^	(N = 35)		(N = 65)		
No therapy	11	31.4	21	32.3	0.403
Chemotherapy	11	31.4	13	20.0	
Chemoradiotherapy	13	37.1	31	47.7	
**Intervention**					
Urgent	60	70.6	52	61.2	0.257
Elective	25	29.4	53	38.8	
**Length of resection** (cm) (mean)	27.0	18.0	25.3	18.9	0.609
**Type of stoma**					
Colostomy	51	60.0	45	52.9	0.439
Ileostomy	34	40.0	40	47.1	
**High output stoma at admission**	3	3.5	7	8.2	0.328
**Hospital stay after surgery** (days) (median)	23	18.5	9	8.5	< 0.001

^a^The population of cancer patients (having received treatment or not) is 35 in the retrospective cohort and 65 in the prospective cohort.

### HOS-related readmissions

#### Twelve-month follow-up

In the retrospective study, 65.9% of patients (n = 56) completed the 12-month follow-up. Twenty per cent (n = 17) died before completing follow-up and 14.1% (n = 12) underwent surgery to restore intestinal continuity.

In the prospective study, 80% of patients (n = 68) completed the 12-month follow-up. 8.2% (n = 7) died before completing follow-up and 11.8% (n = 10) underwent surgery to restore intestinal continuity.

During the follow-up period, a significant difference for HOS-related readmissions was observed between both cohorts, with readmission rates of 28.6% (n = 16) vs 10.3% (n = 7) in the retrospective and prospective cohorts, respectively (Chi-square test; *p* = 0.011; OR: 2.776 [95% CI 1.229–6.269]).

The results of the multivariate analysis are shown in Table 2. ORs and their corresponding confidence intervals are provided for the different unbalanced variables found in the prior cohort analysis. In the retrospective cohort, the OR for HOS-related readmissions at 12 months, adjusted for sex, underlying disease, and length of hospital stay following surgery (days) was 8.872 (95% CI 2.350–33.498) (*p* < 0.01). The Hosmer & Lemeshow test was 0.478.

**Table 2:** Multivariate analysis.

Variables	β	*p*	Odds ratio	95% CI
**Cohort**				
Prospective			1	
Retrospective	2.183	0.001	8.872	(2.350–33.498)
**Sex**				
Male			1	
Female	− 0.209	0.696	0.811	(0.284–2.315)
**Underlying disease**				
Malignant				
Benign	− 0.612	0.280	0.542	(0.179–1.645)
**Hospital stay after surgery (days)**	− 0.006	0.647	0.994	(0.966–1.022)
**Dyslipidaemia**				
Absent				
Present	1.484	0.016	4.413	(1.322–14.733)

### Nutritional parameters and nutritional diagnosis

#### Changes in nutritional parameters

At visit 1, a pronounced change between usual weight (76.41 ± 14.4 kg) and current weight was recorded, with a mean percentage of variation (mean ± SD) of (-) 7.85 ± 7.99. Table 3 shows mean weight and body mass index (BMI) at visits 1 and 2. A significant weight gain was recorded between visit 1 and visit 2, with a mean percentage of variation (mean ± SD) of 1.38 ± 5.53.

**Table 3:** Weight and BMI variation (N = 77).

	Visit1 (mean ± SD)	Visit2 (mean ± SD)	Difference of the means (visit 1–visit 2)	*p* value
Weight (kg)	70.49 ± 13.83	71.58 ± 13.90	1.08 (95% CI 0.03, 2.14)	*p* < 0.05
Body mass index (BMI)	25.38 ± 4.50	25.73 ± 4.30	0.36 (95% CI − 0.01, 0.73)	*p* = 0.06

#### Dietary progression and nutritional status

Of the 72 patients who completed follow-up, 84.7% (n = 61) either maintained a complete diet (n = 32) or improved their dietary pattern during follow-up compared to baseline (n = 29), whereas 11.1% (n = 8) did not experience dietary progression because a low residue diet was maintained and in 4.2% (n = 3) the dietary pattern worsened because diet restrictions were required.

In the initial nutritional assessment during the follow-up consultation, 34.1% of patients (n = 29) had a good nutritional status, 15.3% (n = 13) were at nutritional risk, and 50.5% (n = 43) had some degree of protein-calorie malnutrition: 17.6% mild, 24.7% moderate, and 8.2% severe. A statistically significant improvement was observed in the second evaluation among 34 of the 56 patients (61%) who, in the first evaluation, had been considered liable to improve (*p* = 0.001; McNemar test).

### Cost analysis

*In the retrospective cohort*, the distribution of HOS-related readmissions (n = 16) per medical specialty was: Internal Medicine Unit 56.3% (n = 9), Surgery Unit 31.2% (n = 5), Emergency Unit 12.5% (n = 2). Overall length of hospital stay (days) was 73 days in the Internal Medicine Unit, 52 days in the Surgery Unit was , and 3 days in the Emergency Unit. Based on the cost of stay/day in each unit, the total cost of HOS-related readmissions was €46,397.

*In the prospective cohort*, the distribution of HOS-related readmissions (n = 7) per medical specialty was: Internal Medicine Unit 57.1% (n = 4), Surgery Unit 42.9% (n = 3). Overall length of hospital stay was 24 and 31 days in the Internal Medicine Unit and Surgery Unit, respectively. Based on the cost of stay/day in each unit, the total cost of HOS-related readmissions was €20,746.

In addition, the follow-up nutrition consultations of the 85 patients included in this cohort, taking into consideration both consultations, entailed a total cost of €1,479. Therefore, the total cost associated with the prospective study was €22,225.

### Economic impact assessment

Based on the estimation of the cost associated with HOS-related readmissions in both cohorts, the economic impact assessment of the intervention led to a total saving of €24,175, with a saving of €284 per patient.

## Discussion

Our findings show that the implementation of a nutrition consultation after hospital discharge is cost-effective as it reduces the rate of HOS-related readmissions and has a lower associated cost. The high readmission rates associated with clinical complications occurring in ostomy patients, such as HOS-related dehydration and renal failure among others, are an indication of the need for developing interventions aimed at reducing their incidence and improving their management in order to ameliorate these patients’ health status and reduce healthcare costs^3,6^. Several studies have reported that there is a need to implement procedures ensuring patients are appropriately monitored by providing tools and information on the prevention and management of these complications^19,20^. Although various studies have suggested strategies to be implemented, this is the first study to demonstrate clear benefits resulting from the implementation of a nutritional intervention in this type of patients.

Messaris et al. recorded a 17% readmission rate in ostomy patients following hospital discharge, with HOS-related dehydration as the major cause for readmission^21^. In our study, the rate of HOS-related readmissions in the non-interventional group was much higher: 28.6%. This figure coincides with other authors’ results describing rates of up to 30%, with high output-related dehydration and renal failure as the most common causes: up to 42% and 20%, respectively^6,8^. In contrast, after the implementation of the follow-up consultation following hospital discharge in our prospective study, a significantly lower HOS-related readmission rate was obtained.

Colorectal cancer and inflammatory bowel disease account for the highest percentage of causes associated with surgical interventions^1,8,10,14^. Although several studies have reported predominantly male populations^2,8,22^, Caricato et al*.*^3^ described populations with a higher percentage of women, similar to that in our retrospective cohort, and did not identified sex as a risk factor associated with the occurrence of complications.

Nagle et al*.*^19^ designed an intervention consisting of an aggressive education process and the scheduling of early postoperative visits which led to a pronounced decrease in readmissions associated with dehydration. However, other published education programmes for ostomy patients have not provided consistent results regarding the impact on the reduction in the rate of readmissions^20^. In our case, we observed a clear improvement in output control resulting in a decrease in hospital readmissions and also in an improvement of the nutritional parameters monitored.

Malnutrition secondary to a HOS can have significant consequences on patient health. In the nutritional assessment performed during consultation, 50% of patients were found to have some degree of malnutrition and 15% were at nutritional risk. These findings are in line with other studies and reveal the need to establish specific nutritional measures to ensure appropriate dietary progression^15^. It has been shown that a poor nutritional status contributes to the development of infections, pressure ulcers, delayed wound healing, low functional performance, higher healthcare resource utilization, longer and more frequent hospital stays, and mortality increase^23^. Although we have an established hospital high output management protocol during hospitalization^21^ and many patients are monitored during their hospital stay by a nutritionist, patients lose weight between hospital discharge and their first nutrition consultation. This leads us to believe that patients leave hospital without sufficient knowledge about dietary progression or individual nutritional objective.

After the first nutrition consultation there was a reduction in weight loss, with a modest weight gain observed at the second consultation. Early detection of poor nutritional status during the first assessment, together with the monitoring of the dietary progression following hospital discharge and the design of individualized nutritional support plans for patients during their immediate postoperative period led to an improved dietary transition and enabled a large percentage of patients to reach their nutritional objectives.

The development and implementation of clinical interventions aimed at improving health results should include an assessment of the impact of such measures on healthcare resources^9,10^. Recent publications on hospital readmissions of ostomy patients have described significantly longer hospital stays for patients developing a high output^6,8^. In our case, the total length of hospital stay was longer in the retrospective cohort and, together with the higher number of readmissions recorded, resulted in a much higher total calculated cost compared with the prospective study. Based on the costs of the follow-up consultation that was specifically designed for the prospective study and the results obtained following its implementation, involving a decrease in HOS-related readmissions, the intervention was cost-effective.

## Conclusions

Our study has several limitations, such as the heterogeneity of the study populations, the relatively small number of patients enrolled and the high number of losses to follow-up mostly due to restoration of intestinal continuity, a common surgery in this group of patients. Furthermore, the analysis of the overall cost of readmissions did not include the social cost associated with the loss of patient productivity, which could be of interest when assessing the related economic impact. However, despite these limitations, our results reveal that there is a positive relationship between the intervention performed –based on the nutrition consultation– and a reduction in hospital readmissions following discharge. This shows this intervention could constitute a potential cost-effective strategy for the management of healthcare resources in our hospital.

## Appendix

See Table

**Table 4:** High output stoma detection and management.

When to consider that there is a high output stoma?	Output > 2000 ml/day for more than 48 h Output ≥ 1000 ml/day for 3–5 consecutive days
How do I detect a high output stoma?	It is important that you know the volume of the ostomy bag you are using in order to keep track of your stoma output
What should I do if I have a high output stoma?	Reduce the daily total fluid intake: restrict to one litre taken in small amounts throughout the day Try not to drink during meals Use rehydration solutions Avoid herbal teas, coffee, sodas, and juices
Specific nutritional treatment	Avoid fluid intake during meals Increase the salt content of foods in order to promote fluid resorption Insoluble fibre is contraindicated because of the risk of bowel obstruction

4.

## References

[1] Arumugam PJ, Bevan L, Macdonald L, Watkins AJ, Morgan AR, Beynon J, Carr ND (2003). A prospective audit of stomas—Analysis of risk factors and complications and their management. Colorectal Dis..

[2] Li W, Stocchi L, Cherla D, Liu G, Agostinelli A, Delaney CP, Steele SR, Gorgun E (2017). Factors associated with hospital readmission following diverting ileostomy creation. Tech. Coloproctol..

[3] Caricato M, Ausania F, Ripetti V, Bartolozzi F, Campoli G, Coppola R (2007). Retrospective analysis of long-term defunctioning stoma complications after colorectal surgery. Colorectal Dis..

[4] Vergara-Fernández O, Trejo-Avila M, Santes O, Solórzano-Vicuña D, Salgado-Nesme N (2019). Predictors of dehydration and acute renal failure in patients with diverting loop ileostomy creation after colorectal surgery. World J. Clin. Cases.

[5] Mountford CG, Manas DM, Thompson NP (2014). A practical approach to the management of high-output stoma. Frontline Gastroenterol..

[6] Iqbal A, Sakharuk I, Goldstein L, Tan SA, Qiu P, Li Z, Hughes SJ (2018). Readmission after elective ileostomy in colorectal surgery is predictable. JSLS.

[7] Justiniano CF, Temple LK, Swanger AA (2018). Readmissions with dehydration after ileostomy creation: Rethinking risk factors. Dis. Colon. Rectum..

[8] Fish DR, Mancuso CA, Garcia-Aguilar JE (2017). Readmission after ileostomy creation: Retrospective review of a common and significant event. Ann. Surg..

[9] Carvalho DS, Silva AGID, Ferreira SRM, Braga LC (2019). Elaboration of an educational technology for ostomized patients: Peristomal skin care. Rev. Bras. Enferm..

[10] Chen SY, Stem M, Cerullo M (2018). Predicting the risk of readmission from dehydration after ileostomy formation: The dehydration readmission after ileostomy prediction score. Dis. Colon. Rectum..

[11] McDonald, A. Orchestrating the management of patients with high-output stomas. *Br. J. Nurs.* **23**(12), 645-6, 648-9. 10.12968/bjon.2014.23.12.645 (2014). 10.12968/bjon.2014.23.12.645 25039628

[12] Arenas Villafranca, J. J., López-Rodriguez, C., Fontalva, A., Gándara Adán, N., Utrilla Navarro, P. & Abilés, J. High output stomas detection and approach analisys: Still a challenge. Eur. J. Clin. Pharm. ISSN 2385-409X, Vol. 17, Nº. 4, 2015, págs. 3-3.

[13] Baker ML, Williams RN, Nightingale JMD (2010). Causes and management of a high-output stoma. Colorectal Dis..

[14] Schiergens TS, Hoffmann V, Schobel TN, Englert GH, Kreis ME, Thasler WE, Werner J, Kasparek MS (2017). Long-term quality of life of patients with permanent end ileostomy: Results of a nationwide cross-sectional survey. Dis. Colon. Rectum..

[15] de Oliveira AL, Boroni Moreira AP, Pereira Netto M, Gonçalves Leite IC (2018). A cross-sectional study of nutritional status, diet, and dietary restrictions among persons with an ileostomy or colostomy. Ostomy Wound Manag..

[16] Arenas Villafranca JJ, López-Rodríguez C, Abilés J, Rivera R, Gándara Adán N, Utrilla NP (2015). Protocol for the detection and nutritional management of high-output stomas. Nutr. J..

[17] Mueller C, Compher C, Druyan M (2011). A.S.P.E.N. clinical guidelines nutrition screening, assessment, and intervention in adults. JPEN.

[18] Ministerio de Sanidad, Consumo y Bienestar Social de España. Portal Estadístico del Sistema Nacional de Salud - Estadísticas y Estudio- Estadísticas Sanitarias - Índice Nacional de defunciones (mscbs.gob.es). Accessed 27 April 2020 (2020).

[19] Nagle D, Pare T, Keenan E, Marcet K, Tizio S, Poylin V (2012). Ileostomy pathway virtually eliminates readmissions for dehydration in new ostomates. Dis. Colon. Rectum..

[20] Grahn SW, Lowry AC, Osborne MC (2019). System-wide improvement for transitions after ileostomy surgery: Can intensive monitoring of protocol compliance decrease readmissions? A randomized trial. Dis. Colon. Rectum..

[21] Messaris E, Sehgal R, Deiling S, Koltun WA, Stewart D, McKenna K, Poritz LS (2012). Dehydration is the most common indication for readmission after diverting ileostomy creation. Dis. Colon. Rectum..

[22] Kandagatla P, Nikolian VC, Matusko N, Mason S, Regenbogen SE, Hardiman KM (2018). Patient-reported outcomes and readmission after ileostomy creation in older adults. Am. Surg..

[23] Pérez-Llamas F (2012). Risk of desnutrition in the Spanish population. Evaluation of the current situation and need for a nutritional intervention. Med. Clin. (Barc).

